# RNA-Seq analysis reveals critical transcriptome changes caused by sodium butyrate in DN mouse models

**DOI:** 10.1042/BSR20203005

**Published:** 2021-04-09

**Authors:** Hansen Yang, Zheng Zhang, Rui Peng, Luyu Zhang, Handeng Liu, Xinyi Wang, Yiting Tian, Yan Sun

**Affiliations:** 1 Department of Cell Biology and Genetics, Chongqing Medical University, Chongqing 400016, China; 2 Department of Bioinformatics, Chongqing Medical University, Chongqing 400016, China; 3 Experimental Teaching Center, Chongqing Medical University, Chongqing 400016, China

**Keywords:** diabetic nephropathy, long noncoding RNA, RNA sequencing, sodium butyrate

## Abstract

Diabetic nephropathy (DN)—a common complication of diabetes—is the primary cause of end-stage renal disease. Sodium butyrate (NaB) is a short-chain fatty acid (SCFA) that is a metabolic product of intestinal bacterium, and its protective effect on the kidney has been reported in cases of DN. However, its underlying mechanism remains unclear. The aim of the present study was to investigate the effect of NaB on globe transcriptome changes in DN. In our study, 8-week-old male db/db mice suffering from DN were randomly divided into two groups: the DN+NaB group (DN mice treated with NaB, 5 g/kg/day) and the DN group (DN mice treated with saline). Further, normal db/m mice were used as the normal control (NC) group. The blood glucose, body weight, urinary microalbumin and urinary creatinine of mice were measured for all three groups. Whole-transcriptome analysis was performed by RNA sequencing (RNA-Seq) to evaluate the profiling of long non-coding RNAs (lncRNAs) and messenger RNAs (mRNAs). Bioinformatics analysis was performed to predict the potential NaB-related lncRNAs and genes in DN. The expressions of lncRNAs and mRNAs were tested using the quantitative real-time polymerase chain reactions (qRT-PCRs) in renal tissues and mesangial cells treated with NaB. The results of the present study demonstrated that NaB ameliorated renal dysfunction in DN mice. Moreover, RNA-Seq results identified that some lncRNAs and mRNAs were reversely changed in the DN+NaB group in comparison to those in the DN group. Additionally, the integrated co-expression networks of NaB-related lncRNAs revealed that these lncRNAs interacted with 155 key mRNAs. Furthermore, the co-expression network of inflammation-related lncRNAs and mRNAs demonstrated that those reversed lncRNAs and mRNAs also play essential roles in the inflammatory response. In summary, the present study suggests that NaB ameliorates diabetes-induced renal dysfunction and regulates transcriptome changes in DN.

## Introduction

As a common complication of diabetes, diabetic nephropathy (DN) is one of the main causes of end-stage renal disease [1–3]. However, the prevention and treatment of DN still requires research and, to the best of our knowledge, the underlying pathogenesis of DN is unknown.

Emerging evidence has shown that short-chain fatty acids (SCFAs) are widely associated with the development of many diseases [4,5]. SCFAs are the main metabolic products of the bacterial fermentation of the macro-fibrous material that escapes digestion in the upper gastrointestinal tract and enters the colon. Further, 90–95% of the SCFAs in the colon are made up of acetates (60%), propionates (25%) and butyrates (15%) [6,7]. Recently, studies have shown that butyrates, as the important component of SCFAs, play a crucial role in the progression of diseases, including inflammatory dermatoses, autoimmune uveitis, inflammatory bowel disease and DN [8–10]. Roberta et al. demonstrated that sodium butyrate (NaB) prevents the translocation of NF-κB into the nucleus, decreasing oxidative damage, the inflammatory response and tubular damage in patients with diabetes [11,12]. Further, Dong et al. found that Nrf2 plays a key role in the protective effect of NaB against DN, indicating that NaB regulates Nrf2 expression at the transcriptional level, possibly through the inhibition of HDAC activity [13]. These findings suggest that NAB, an SCFA, can prevent the progression of DN by altering the gene-expression profile and signaling pathway. However, the specific mechanism of NaB in DN is still not understood well.

As a technique for detecting the expression level of the transcriptome, RNA sequencing (RNA-Seq) has the advantages of quantitative accuracy, high levels of reproducibility and a wide detection range, and is widely used in transcriptome research [14,15]. In this study, RNA-Seq analysis was performed to determine the expression of profiles of messenger RNAs (mRNAs) and long non-coding RNAs (lncRNAs) in the renal cortex tissue of normal control (NC) mice and db/db DN mice, with or without NaB treatment. The results showed that 180 mRNAs and 17 lncRNAs could be involved in DN in mice treated using NaB. Subsequently, we also constructed the lncRNA–mRNA interaction network to predict the potential lncRNAs and mRNAs associated with the mechanism of NaB for ameliorating DN. GO and KEGG pathway analysis found that NaB may closely related to inflammation, inhibiting the inflammatory response for ameliorating DN. The aim of the study was to provide novel insights into the benefits and potential mechanisms of action of NaB against DN.

## Materials and methods

### Animals

Eight-week-old male BKS.Cg- +Leprdb/+Leprdb/J(db/db) mice and their age-matched heterozygous male littermates (BKS.Cg-m+/+Leprdb/J(db/m) mice) were obtained from the Nanjing Biomedical Research Institute of Nanjing University (Nanjing, China). The db/m mice were used as NC group (*n*=5). The db/db mice were randomly divided into two groups: the DN mice treated with saline (DN group, *n*=5) and the DN mice treated with NaB through intraperitoneal injection (DN+NaB group, 5 g/kg/day, *n*=5). Glucose was measured using a blood-glucose meter (Roche, Sweden); body weight was measured using an electronic weighing scale; urinary microalbumin was detected using a mouse-specific microalbuminuria enzyme-linked immunosorbent assay (ELISA) kit (Jiancheng, Nanjing, China); and urinary creatinine was tested using a creatinine assay kit (Jiancheng, Nanjing, China). After 4 weeks of medication, the mice were anesthetized with 1.5% pentobarbital sodium (Sigma–Aldrich Chemical Company, St. Louis, MO, U.S.A., intraperitoneal injection) and were killed by cervical dislocation. The renal cortices of the mice were harvested and preserved in liquid nitrogen for further RNA-Seq analysis. All animals were maintained under standard specific pathogen-free conditions in the Experimental Animal Center of Chongqing Medical University, China, where the animal work took place. All procedures were conducted following the institutional guidelines for the care and use of laboratory animals at Chongqing Medical University. Ethics approval was obtained from the Ethics Committee of Chongqing Medical University.

### Histological analysis

The mice’s renal cortices were placed in a 4% paraformaldehyde solution for 24 h and embedded in paraffin, followed by sectioning into pieces of 5-µm-thickness. Hematoxylin–Eosin (H&E) and Periodic Acid–Schiff (PAS) staining analyses were performed to assess pathological changes in the renal tissue [16]. The digital images were observed through microscopy (Olympus, Tokyo, Japan).

### Cell culture

The mouse kidney mesangial cell line (MC, SV40-MES14) was cultured in Dulbecco’s modified Eagle’s medium (DMEM) with 10% fetal bovine serum in a 37°C incubator with 5% CO_2_. Furthermore, MCs were stimulated with 5.5 mmol/l glucose and 19.5 mmol/l mannitol (low glucose group; LMC), with 25 mM glucose (high glucose group; HMC) or with 25 mM glucose plus 5 mM NaB (HMC+NaB) for 48 h [17].

### RNA isolation and RNA-Seq

Total RNA was separately extracted from the renal cortices of four mice in the NC group, four mice in the DN group and four mice in the DN+NaB group using TRIzol reagent (Invitrogen, California, U.S.A.). The RNA quality was tested using Bioanalyzer 2200 (Agilent, California, U.S.A.). The cDNA libraries were constructed for each pooled RNA sample using the VAHTS™ Total RNA-Seq (H/M/R), according to the manufacturer’s instructions. The products were purified and enriched by PCR to create the final cDNA libraries and quantified using Agilent 2200 (California, U.S.A.).

### Sequence data analysis

Before read mapping, clean reads were obtained from the raw reads by removing the adaptor sequences—reads with >5% ambiguous bases (denoted as N) and low-quality reads containing more than 20% of bases with qualities of <20. The clean reads were then aligned with the mouse genome (version: GRCh38NCBI) using the HISAT2 [18]. HTseq was used to count gene and lncRNA counts, and the RPKM method was used to determine the gene expression. The DESeq algorithm was applied to filter the differentially expressed genes after an analysis of the significance, *P*-value and false discovery rate (FDR) under the following criteria: log_2_FC > 0.585 or < −0.585, and an FDR < 0.05.

### Kyoto Encyclopedia of Genes and Genomes pathways and gene ontology analysis

Pathway analysis was used to determine the significant pathways of the differential genes according to the KEGG database (http://www.genome.jp/kegg/). Fisher’s exact test was used for selecting the significant pathway, and the threshold of significance was defined by the *P*-value and FDR. Gene ontology (GO) analysis was performed to construct the main function of the differentially expressed mRNAs. Fisher’s exact test was used to identify the significant GO categories and FDR was used to correct the *P*-values. Cytoscape was used to construct the pathway network.[19]

### Co-expression network of lncRNAs and mRNAs

To predict the functions of NaB-related lncRNAs based on the annotations of the co-expressed mRNAs, a co-expression network between the two significant series-cluster profiles (profiles 1 and 2) of lncRNAs and differentially expressed mRNAs associated with NaB was constructed [20]. The Pearson correlation coefficient, calculated using the R-value between lncRNAs and mRNAs, should be greater than 0.99. Cytoscape was used to create this co-expression network.

### Quantitative real-time polymerase chain reaction analysis

TRIzol reagent (Invitrogen, California, U.S.A.) was used for the total RNA extraction from the renal cortices of mice (*n*=4 for the NC, DN and DN+NaB groups) and three groups of MCs (triplicate experiments for the LMC, HMC and HMC+NaB group). The Prime Script RT Reagent Kit (Takara Bio, Dalian, China) was used for cDNA construction through the reverse transcription reaction. Quantitative real-time polymerase chain reaction analysis (qRT-PCR) was performed with TB Green® Premix ExTaq™ II (Takara Bio) using the CFX Connect Real-Time PCR Detection System (Bio-Rad, Hercules, CA). The 2^−ΔΔ*C*_t_^ method was used to calculate the relative fold changes of RNA expression, and β-actin was used for normalizing the data. The primer sequences are provided in Supplementary Table S1. All experiments were performed three times.

### Statistical analysis

All values are given as mean ± SEM, and statistical analyses were performed using one-way analysis of variances with Tukey’s multiple comparison tests. *P*-values below 0.05 were considered to be statistically significant differences. GraphPad Prism 7.0 (GraphPad Software, San Diego, U.S.A.) was used to analyze the data.

## Results

### NaB ameliorates diabetes-induced renal dysfunction

**Figure 1:**
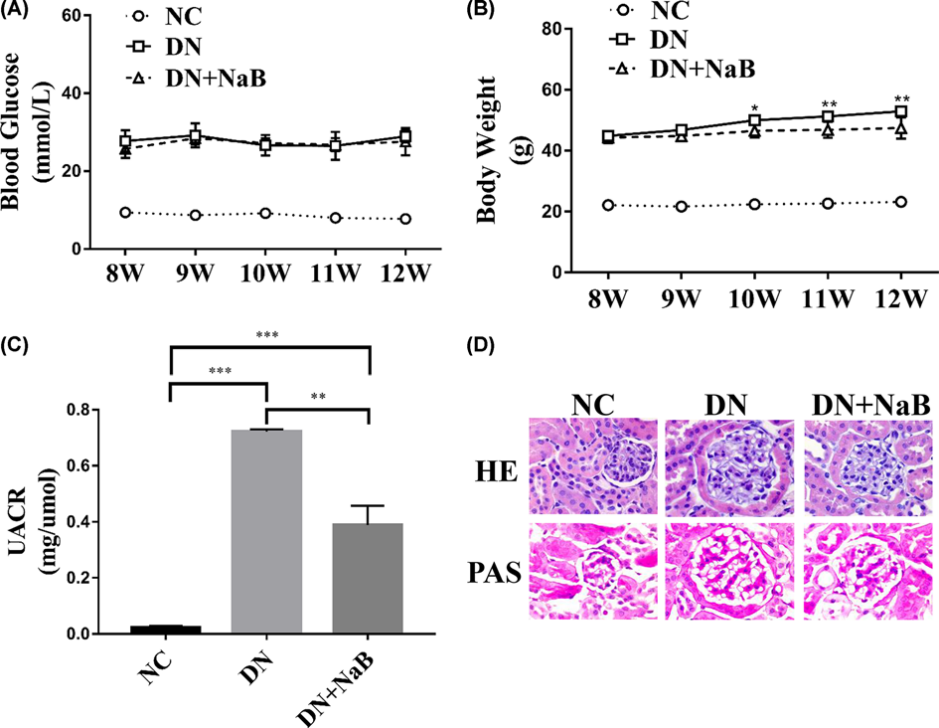
NaB ameliorates diabetes-induced renal dysfunction in DN mice (**A**) Significant differences in body weight between the DN (db/db DN mice group, *n*=5) and DN+NaB (db/db DN mice treated with NaB group, *n*=5) groups. (**B**) There are no significant differences between the DN and DN+NaB groups in terms of fasting blood glucose, *P<0.05, **P<0.01. (**C**) Significant differences exist between the DN and DN+NaB groups in terms of urinary microalbumin excretion in 12 weeks. All values are represented as mean ± SD, ***P<0.001. (**D**) H&E and PAS staining show glomerular and tubular injuries in the NC, DN and DN+NaB groups (magnification ×400).

In comparison to db/m mice (NC group), all diabetic mice showed a significant increase in blood glucose and body weight levels at 8 weeks of age (Figure 1A,B). At 12 weeks of age, the rate of weight gain for diabetic mice treated with NaB (DN+NaB group) was slower than that of diabetic mice (DN group) (Figure 1A), while there was no significant difference in blood glucose levels (Figure 1B). In addition, the urine albumin-to-creatinine ratio (UACR) levels were reduced in the DN+NaB group (Figure 1C). To further investigate the influence of NaB on diabetes-induced renal pathological changes in diabetic mice, H&E staining and PAS staining were performed for morphological analysis (Figure 1D). These data showed that the glomerular area and mesangial matrix expansion were significantly ameliorated in the DN+NaB group in comparison to the DN group. The above results indicate that NaB could have a protective effect against diabetes-induced renal dysfunction.

### NaB regulates mRNA and lncRNA expression in diabetic mice’s kidneys

**Figure 2:**
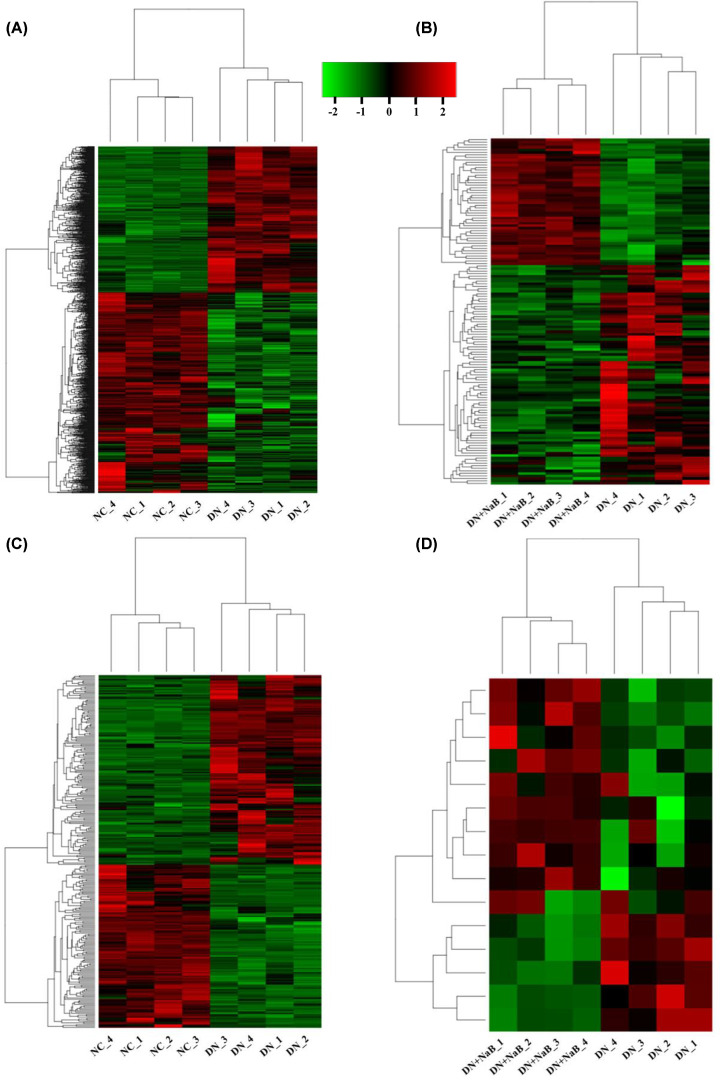
Heatmap showing the expression profiles of mRNAs and lncRNAs (**A,B**) Differentially expressed mRNAs in NC vs DN mice (A) and the DN vs DN+NaB groups (B) (log_2_FC > 0.585 or log 2 FC < −0.585, FDR < 0.05, *n*=4). (**C,D**) Differentially expressed lncRNAs in NC vs DN mice (C) and the DN vs DN+NaB groups (D) (log_2_FC > 0.585 or log_2_FC < −0.585, FDR < 0.05, *n*=4). Hierarchical clustering and heat mapping are performed to investigate the differences in genes among various groups. Each row represents a single gene and each column represents a single tissue sample.

To understand the underlying molecular mechanisms of NaB in improving DN, we performed RNA-Seq to reveal differently expressed mRNAs and lncRNAs in kidney tissue from the NC, DN and DN+NaB groups (*n*=4 for each group). After normalizing and filtering all RNA-Seq data, hierarchical clustering and heatmaps showed the expression changes between the NC, DN and DN+NaB groups (log_2_FC > 0.585 or < −0.585, FDR < 0.05). When comparing the NC and DN group, 1252 mRNAs (528 up-regulated and 724 down-regulated) and 277 lncRNAs (149 up-regulated and 128 down-regulated) were differentially expressed in the kidney tissue (Figure 2A,C). When comparing the DN and DN+NaB groups, 137 mRNAs (50 up-regulated and 87 down-regulated) and 15 lncRNAs (10 up-regulated and 5 down-regulated) were differentially expressed in the kidney tissue (Figure 2B,D).

### Reversely changed mRNAs following NaB treatment were enhanced in terms of certain biological functions

**Figure 3:**
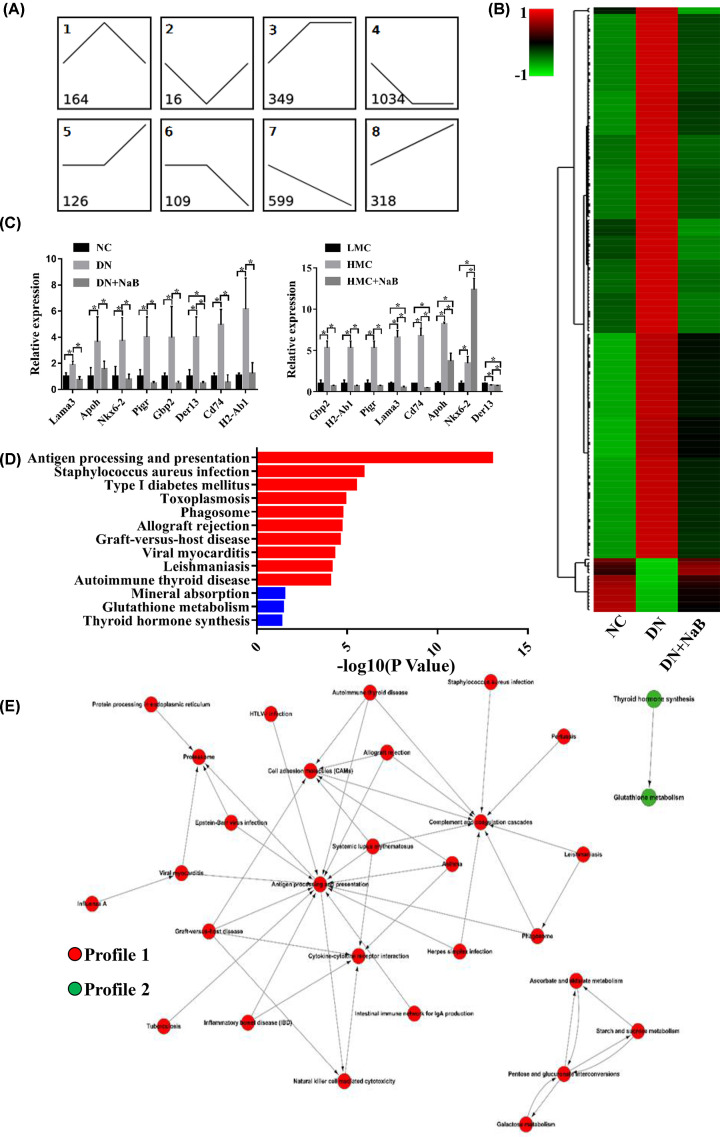
Reversely changed mRNAs following NaB treatment are enhanced in terms of certain biological functions (**A**) Model-based expression profiles (profiles 1–8) of mRNAs expressed differentially for the NC, DN and DN+NaB groups. The number at the top-left denotes the profile ID, and the number at the bottom-left denotes the mRNA count. (**B**) Hierarchical clustering and heatmaps showing those reversed mRNAs (profiles 1 and 2) differentially expressed in the NC, DN and DN+NaB groups. (**C**) The eight reversed mRNAs detected by qRT-PCR in DN mice and mice mesangial cells. The histogram at the top shows the mRNA expression in the NC, DN and DN+NaB groups (*n*=4 in each group). The histogram at the bottom represents the mRNA expression in mice mesangial cells (MC) treated with 5.5 mM glucose (LMC), 25 mM glucose (HMC) or HMC treated with 5 mM NaB (HMC+NaB). The data are representative of three independent experiments. *P<0.05. (**D**) KEGG pathway enrichment analysis of reversed mRNAs depicting the top 13 significant (*P*<0.05) pathways. The red row represents the profile 1-type mRNA enriching pathway, and the blue row represents the profile 2-type mRNA enriching pathway. (**E**) Pathway-Act-Network according to the overlaps and links between associated molecules in the top 13 significant canonical pathways. The red nodes indicate that the signaling pathway with profile 1-type mRNA enrichment and the green nodes indicate the signaling pathway with profile 2-type mRNA enrichment.

A total of 2715 mRNAs were classified into eight profiles (Figure 3A). Among them, 180 mRNAs were reversely changed following NaB treatment (profiles 1 and 2). All of these 180 mRNAs meet the following criteria: (1) in comparison to NC mice, they were significantly altered in DN mice’s kidneys. (2) Their expression was reversed in DN+NaB mice. With these criteria, there was an increase in 164 mRNAs in the DN group, which returned to the control level in the DN+NaB group in profile 1. On the other hand, profile 2 included 16 mRNAs that were down-regulated in the DN group, which then returned to the baseline value in the DN+NaB group. Hierarchical clustering and heatmaps were conducted to reveal the series of changes of the 180 mRNAs in the NC, DN and DN+NaB groups (Figure 3B). Furthermore, to validate the RNA-Seq results, eight reversed mRNAs were selected randomly to be measured by qRT-PCR in the renal tissue of mice and mouse mesangial cells. All results are shown in Figure 3C. The qRT-PCR data of Lama3, Apoh, Nkx6-2, Pigr, Gbp2, Der13, Cd74 and H2-Ab1 were increased in the DN group and down-regulated in the DN+NaB group, which verified the accuracy of RNA-Seq in mice. Further, the mRNA levels of Lama3, Apoh, Pigr, Gbp2, Cd74 and H2-Ab1 were also increased in MCs cultured with high glucose (HMC) and down-regulated in HMC treated with NaB (HMC+NaB). Most of these results were consistent with the RNA-Seq *in vivo* and *in vitro* (Figure 3C). To verify the functional analysis of these mRNAs, KEGG pathway enrichment has been demonstrated [21]. Our data showed the top 10 pathways related to profile 1 mRNAs and three pathways significantly related to profile 2 mRNAs (Figure 3D). All significant pathways of reversed mRNAs are shown in Supplementary Table S2. Pathway enrichment analysis revealed that the antigen processing and presentation of staphylococcus aureus infection were the main pathways for the profile 1 mRNAs. Further, the significantly enriched pathways for profile 2 mRNAs were mineral absorption, glutathione metabolism and thyroid hormone synthesis. To explore the deep interactions among these significant pathways for reversely changed mRNAs, Pathway-Act-Network analysis was performed (Figure 3E). The results of this analysis revealed that most pathways were associated with antigen processing and presentation, These findings suggest that NaB treatment ameliorates the development of DN by modulating mRNAs and their downstream pathways in the kidneys of DN mice.

### LncRNAs are reversely changed following NaB treatment in the DN mouse model

**Figure 4:**
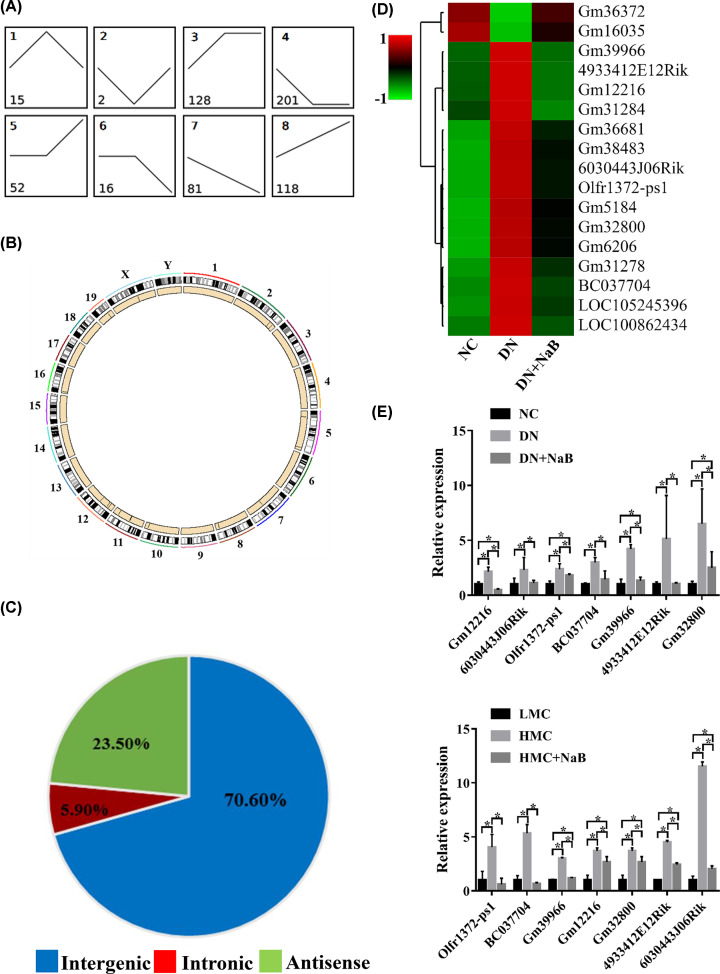
LncRNAs are reversely changed following NaB treatment in the DN mouse model (**A**) Model-based expression profiles (profile 1–8) of lncRNAs expressed differentially for the NC, DN and DN+NaB groups. Numbers in the profiles indicate profile IDs (top-left) and lncRNA counts (bottom-left). (**B**) Circos plot showing reversed lncRNAs in mouse chromosomes. The outermost layer of the circos plot is the chromosome map of the mouse genome—the larger inner circle indicates the reference genome localization. The smaller inner circle indicates the outline of the chromosome, and the position of the reversed inverted lncRNA is marked using a black line. (**C**) Pie charts showing the proportions of three types of reversed lncRNAs (profiles 1 and 2) in the NC, DN and DN+NaB groups. (**D**) Hierarchical clustering and heatmaps showing the reversed lncRNAs (profiles 1 and 2) differentially expressed in the NC, DN and DN+NaB groups. (**E**) The seven reversed lncRNAs detected using qRT-PCR in DN mice and mice mesangial cells. The top shows the expression of reversed lncRNAs in the NC, DN and DN+NaB groups (*n*=4 in each group) and the bottom shows the expression of reversed lncRNAs in the LMC, HMC and HMC+NaB groups (triplicate experiments). *P<0.05.

A total of 613 lncRNAs were classified into eight profiles (Figure 4A). Using the criteria set out in the above paragraph, 17 lncRNAs were reversely changed following NaB treatment (profile 1 included 15 lncRNAs and profile 2 had two lncRNAs). These lncRNAs are mainly distributed in chromosomes 2, 5, 7, 8, 10, 11, 12, 16, 19 and sex chromosomes X (Figure 4B). These reversed lncRNAs, in line with their genomic loci, were classified into three categories: 73.33% were intergenic, 20.00% were antisense and 6.67% were intronic (Figure 4C). Hierarchical clustering and heatmaps (Figure 4D) were constructed, which revealed differences in expression among the NC, DN and DN+NaB groups. Similarly, seven reversed lncRNAs were randomly selected and verified using qRT-PCR in the renal tissue of mice and MCs. As shown in Figure 4E, qRT-PCR data of Gm12216, 6030443J06Rik, Olfr1372-ps1, BC037704, Gm39966, 4933412E12Rik and Gm32800 were increased in the DN group and down-regulated in the DN+NaB group, which verified the accuracy of RNA-Seq in mice. Further, seven lncRNAs levels were also increased in the HMC group and down-regulated in the HMC+NaB group. The results demonstrated that the expressing tendency of the seven lncRNAs tested by qRT-PCR was consistent with that found through RNA-Seq (Figure 4E) analysis *in vivo* and *in vitro*.

### Co-expression network of reversed lncRNA–mRNA and functional predictions

**Figure 5:**
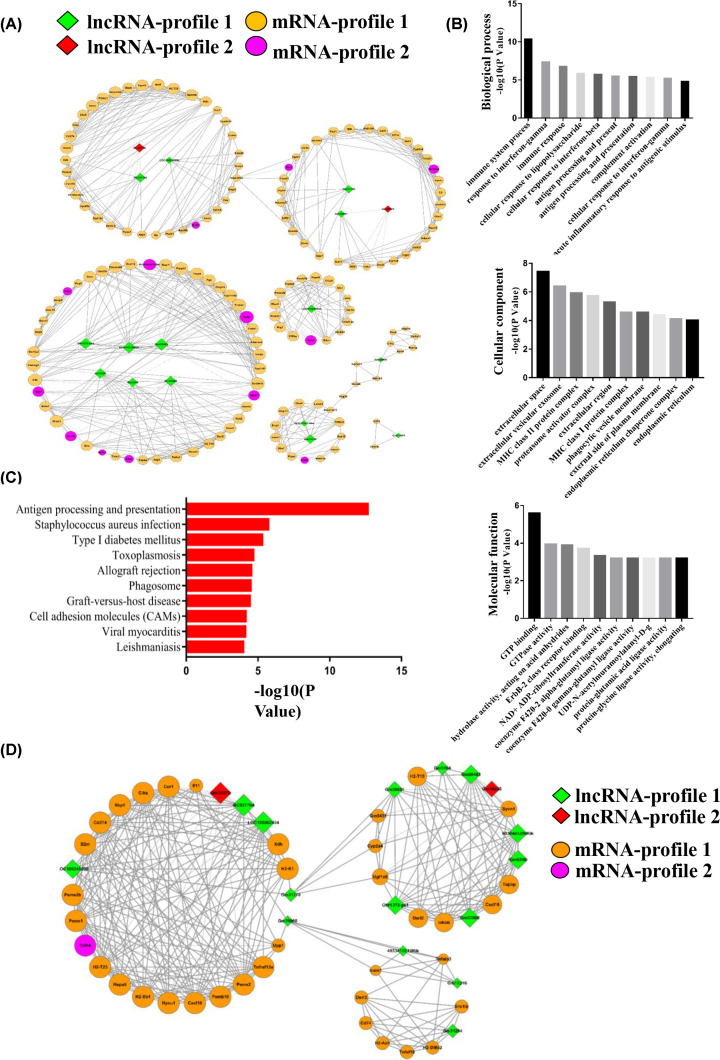
Co-expression network analysis of reversely expressed lncRNAs with their associated mRNAs (**A**) Co-expression relationships between 17 reversely expressed lncRNAs and their 155 associated mRNAs. The green nodes indicate profile 1-type lncRNAs, the red nodes indicate profile 2-type lncRNAs, the yellow nodes indicate profile 1-type mRNAs and the pink nodes indicate profile 2-type mRNAs. (**B**) GO annotation of co-expression mRNAs with top ten –log_10_(p-value) for biological processes, cellular components and molecular functions. (**C**) KEGG pathway enrichment analysis of co-expression mRNAs with top ten −log(*P*-value). (**D**) Co-expression relationships between 17 reversely expressed and inflammation-related lncRNAs and their 37 associated mRNAs. The green nodes indicate profile 1-type lncRNAs, the red nodes indicate profile 2-type lncRNAs, the yellow nodes indicate profile 1-type mRNAs and the pink nodes indicate profile 2-type mRNAs.

To predict the potential functioning of the reversed lncRNAs, a reversed lncRNA–mRNA co-expression network was created [20,27]. A total of 17 reverse lncRNAs and their possible 155 target mRNAs were calculated using the Pearson correlation coefficient. Seven hundred and twenty-six pairs of lncRNAs and mRNA relationships (R > 0.99) were imported into Cytoscape to construct the co-expression core network (Figure 5A). GO analysis of significantly reversely expressed mRNAs can reveal the roles of their interactional lncRNAs (Figure 5B). Our data showed that the reversed mRNAs associated with biological processes were immune response, antigen processing and presentation, and acute inflammatory response to antigenic stimulus. Subsequently, KEGG pathway analysis was performed among these mRNAs to verify the possible mechanism of NaB that regulates DN (Figure 5C). As shown in Figure 5C, the antigen processing and presentation was the main pathway of the top 10 significant pathways, and was also associated with inflammation [28]. It is important to note that phagosome and cell-adhesion molecules were related to inflammation, which were also significant pathways. All significant pathways of mRNAs associated with reversed lncRNAs are provided in Supplementary Table S3, and 37 mRNAs were clustered in inflammation-related pathways. To further understand the inflammatory relationship between the reversed lncRNAs and mRNAs, subnetworks of the co-expression network were constructed using 17 lncRNAs and 37 mRNAs, which participated in the inflammatory pathways (Figure 5D).

## Discussion

Butyrates are part of the SCFA family, which has been reported to be involved in a number of diseases, including uveitis, inflammatory bowel disease and DN [8,9,11]. A study by Dong et al. showed that NaB activated Nrf2, which inhibits HDAC and ameliorates DN-induced renal oxidative damage, apoptosis, inflammation, fibrosis, pathological changes and renal dysfunction [13]. It provided some evidence for NaB’s protective effect against DN. However, the underlying regulatory mechanism of NaB in DN is not fully understood yet. In the present study, naturally developed DN in mice with genetic defects in the leptin receptor (db/db) is a well-established model for type 2 diabetes, obesity and insulin resistance. At the same time, compared with control mice, the level of inflammation-related factors in this model mouse is significantly higher, so it is often used as a model mouse to study inflammation in DN. We conducted RNA-Seq to observe all the transcriptome changes in the renal tissue of db/db DN mice treated with NaB. Through the following bioinformatics analysis, we filtered 17 lncRNAs and 180 mRNAs that were expressed reversely after NaB treatment in db/db DN mice. Furthermore, KEGG and Pathway-Act-Network analysis showed that the 180 reversed mRNAs were enriched in the pathways related to antigen processing and presentation, cell-adhesion molecules, proteasomes, cytokine–cytokine receptor interactions, TNF signaling etc., which were widely known as the immune-related pathways and played a critical role in the inflammatory response [22], and these pathways were reported to be associated with inflammation [23–26]. The genes identified in these pathways involved a wide range of classical biological processes regarding the immune response and inflammation in DN [29–31]. It was found that three genes (Psme1, Psme2 and Psme2b)—as subunits of proteasome activator (PA28) complexes—were down-regulated by NaB in DN, and took part in antigen presentation and proteasomes. The activation of PA28 proteins could exacerbate the pathogenesis of DN, and PA28 activated the 20S proteasome by either facilitating substrate entry or product exit [32,33]. We have also found that Lmp7 (a subunit of the 20S proteasome) and NF-κB (p65/p50) activation led to enhanced pro-inflammatory molecule expression and activation in DN [34,35]. Based on these analyses, it can be stated that NaB reversed PA28 against inflammatory effects in DN. On the other hand, the genes involved in cytokine–cytokine receptor interaction, cell adhesion and TNF signaling pathways included Cxcl16, Tnfrsf12a, Tnfsf10, Tnfaip3 and Icam1, which were also inhibited by NaB in DN. These reversed genes confirmed that inflammation-related molecules and pathways are critically involved in the progression of DN [29,36–40]. A recent study that administered NaB to db/db mice significantly decreased the intercellular adhesion molecule (ICAM-1) in their gut [41]. Current research mainly focuses on the anti-inflammatory mechanisms of NaB on the receptor NF-κB signaling pathway or NLRP3 inflammasome [42,43]. As the results show, most reversed genes were closely associated with the inflammatory response and pathways. The RNA-Seq analysis provides novel molecules and pathways to support NaB in an anti-inflammatory role during the progression of DN.

It has been verified that lncRNAs play a key role in the pathogenesis of DN. As reported in previous studies, lncRNAs can function as regulators in diabetes-induced renal inflammation and histological changes [44,45]. In our study, 17 lncRNAs were identified that were significantly reversely expressed in the renal tissue of the db/db DN mice following NaB treatment. These 17 lncRNAs can function in *cis*- or *trans*-configurations to affect their target protein-coding genes. However, their specific effects on the process of NaB improving DN conditions are unknown. Thus, constructing a lncRNA–mRNA co-expression core network is a common method to predict the functioning of lncRNAs. There were 17 lncRNAs and 155 mRNAs involved in the co-expression network. GO analysis showed that these mRNAs were enriched in categories related to immune response, antigen processing and presentation, cellular response to lipopolysaccharides, acute inflammatory response to an antigenic stimulus etc. These categories are also associated with renal inflammation [45,46]. In addition, we revealed a large group of protein-coding genes that could be possibly affected in *cis*- or *trans*-configurations by the corresponding lncRNAs. For example, Cd74 (potential target of lncRNA Gm31284) was considered to regulate renal inflammation in kidney disease. Cd74 could be activated using the macrophage migration inhibitory factor (MIF) to increase inflammatory cytokines in podocytes and tubular cells, along with proliferation in glomerular parietal epithelial cells and cyst cells. Further, it could interfere with MIF/CD74 signaling and CD74 deficiency, which can have protective effects against crescentic glomerulonephritis [47,48]. In our study, the expression of Gm31284 was reversed by NaB. Based on these analyses, it can be suggested that NaB acts against the inflammatory effects of DN by reversing Gm31284 and its target mRNA Cd74. Further, Spp1—a type of osteopontin known to be a proinflammatory cytokine—has been identified as a key component of cell-mediated immunity [49]. It has been determined that osteopontin can be inhibited by the liver X receptor to relieve inflammation caused by DN [50]. In our study, the level of Spp1 was closely related to the lncRNA Gm31278, and was down-regulated by NaB. Therefore, it is also suggested that NaB can reverse the expression of Gm31278 to inhibit Spp1 and reduce inflammation in DN. Furthermore, oxidative stress has been shown to be closely related to inflammatory response, which is highly involved in the progression and development of DN [51]. XDH expression can be suppressed by gene silencing or small chemical inhibitors, leading to a reduction in overall reactive oxygen species (ROS) levels, which can alleviate the oxidative stress caused by ROS [52]. In our data, XDH—as a target of LncRNA BC037704—was down-regulated by NaB in DN. Based on these analyses, it is suggested that NaB may reverse the expression of LncRNA BC037704 and its target mRNA XDH, working against oxidative stress and the inflammatory effects of DN.

Previous studies have shown that NaB could reduce inflammation in DN, but the relationship between lncRNAs and inflammation is still unclear. Thus, the 17 lncRNAs and 37 mRNAs related to inflammation were used to construct a lncRNA–mRNA co-expression core network. In this network, lncRNA BC037704 and Gm31284 regulated mRNA Xdh and Cd74, respectively, which have been demonstrated to play roles in the inflammatory response. Further, Icam 1, regulated by Gm12216 and Gm39966, was thought to play an important role in T-cell migration into the kidney. The aberrant migration of T cells into tissues contributes to the development of most chronic inflammatory diseases, including DN. The inflammation-related lncRNA–mRNA co-expression networks showed that the reversely expressed lncRNAs could play essential roles in ameliorating inflammation, which is consistent with the anti-inflammatory effect of NaB.

Based on RNA-Seq and bioinformatics analysis, we discussed the possibility that NaB could improve DN by acting on the transcriptome. The accuracy of sequencing results was verified through qRT-PCR. However, more in-depth research into its mechanism is still required. In addition, only animal models were used in the present study. These are some issues that will be taken into consideration in future research.

In summary, we conducted RNA-Seq analysis to identify a group of reversely expressed genes following NaB treatment, and the subsequent bioinformatics analysis demonstrates that these changes may affect renal inflammation. Among these genes, we discovered that lncRNAs are closely associated with renal inflammation. These results suggest that NaB could improve DN by altering lncRNA expression in mice kidneys, and provide the basis for future research.

## Supplementary Material

Supplementary Table S1-S3

## Data Availability

The data used to support the findings of the present study are available from the corresponding author upon request.

## Abbreviations

DN: diabetic nephropathy
FDR: false discovery rate
GO: gene ontology
HDAC: Histone deacetylases
HTseq: High-Throughput sequencing
H&E: Hematoxylin–Eosin
lncRNA: long non-coding RNA
MIF: migration inhibitory factor
mRNA: messenger RNA
NaB: sodium butyrate
Nrf2: Nuclear factor E2-related factor 2
qRT-PCR: quantitative real-time polymerase chain reaction
RNA-Seq: RNA sequencing
ROS: reactive oxygen species
RPKM: reads per kilobase of transcript per million reads mapped
SCFA: short-chain fatty acid
XDH: xanthine dehydrogenase
